# Problematic and adaptive eating in people with obesity after a DBT-based skills training intervention: 3- and 8-month follow-up and mediation analysis

**DOI:** 10.1186/s41155-019-0116-5

**Published:** 2019-01-15

**Authors:** Lucas André Schuster de Souza, Ana Carolina Maciel Cancian, Thiago Gomes de Castro, Margareth da Silva Oliveira

**Affiliations:** 1 Pontifícia Universidade Católica do Rio Grande do Sul (PUCRS), Avenida Ipiranga, 6681, Prédio 11, 9° Andar, Sala 927, Partenon, Porto Alegre, RS 90619-900 Brazil; 2 Universidade Federal do Rio Grande do Sul, Rua Ramiro Barcelos, 2600-Sala 123, Rio Branco, Porto Alegre, RS 90035003 Brazil

**Keywords:** Dialectical behavior therapy, Emotional eating, Mindful eating, Intuitive eating, Binge eating, Obesity

## Abstract

**Background:**

Dialectical behavior therapy conceptualizes problematic behaviors as attempts to regulate emotions that occur when the individual lacks effective skills with which to manage his or her emotions and cope with distress. Problematic eating behaviors, e.g., binge and emotional eating, may serve to alleviate aversive emotional states, being highly associated with overweight and obesity. Dialectical behavior therapy skills training has been proven effective in reducing binge eating in several clinical studies. However, few studies reveal the effects of DBT on adaptive eating behaviors or the stability of outcomes.

**Objectives:**

This study aimed to test the effect of a brief DBT-based skills training intervention, and the stability of outcomes at 3- and 8-month follow-ups.

**Methods:**

Self-report measures of binge eating, emotional eating, intuitive eating, and mindful eating were taken on 5 timepoints before and after a 10-session DBT skills training intervention (2 baseline measures, 1 post-test, and 2 follow-ups). Data were analyzed using a mixed-model intention-to-treat approach and mediation analysis was conducted with path analysis.

**Results:**

After the intervention, intuitive eating and mindful eating scores were significantly higher than before the intervention, while emotional eating and binge eating scores were lower. The results remained stable during the follow-up period, with minor fluctuations and small trends towards returning to baseline values for binge eating and emotional eating. Mindful eating partially mediated the improvements in all outcomes.

**Limitations:**

Given that results are entirely based on self-report measures and that some instruments showed poor reliability, in addition to the high attrition rates, the results should be interpreted as preliminary.

**Conclusions:**

The results provide evidence that a brief DBT intervention is effective not only in reducing problematic eating but also in increasing adaptive eating, achieving reasonably stable results. Also, the mediation analysis results support the hypothesis that mindful eating partially explains the effects of the intervention on binge and emotional eating. Future research should address the limitations of this study by investigating a more diverse sample, triangulating different measurement strategies, and including other putative mediators.

## Background

Dialectical behavior therapy [DBT] was originally developed to treat individuals with pervasive emotional dysregulation. However, its modularity facilitated adapting the treatment to other populations, with skills modified to meet diverse clinical needs, such as those of individuals with problematic eating behaviors (Linehan and Wilks, 2015; Safer, Telch, and Chen, 2009). The rationale behind these adaptations is the conceptualization of problematic behaviors as attempts to regulate intense emotional states, combined with a lack of effective skills with which to manage emotions and tolerate distress (Linehan, 1993). The affect regulation model proposes that binge eating behaviors alleviate unpleasant emotions and are thus negatively reinforced in the long term (Polivy and Herman, 1993).

Dialectical behavior therapy skills training flourished as a standalone treatment for a vast range of clinical conditions, including binge eating and other eating problems (Valentine, Bankoff, Poulin, Reidler, and Pantalone, 2015). Studies of DBT skills training adapted to problematic eating behaviors started in 2001, with the studies conducted by Safer et al. (2001) on bulimic women and by Telch et al. (2001) on women with binge eating. Both studies compared a skills training group with waitlist control conditions and found improvements regarding binge/purge behaviors (Safer, Telch, and Agras, 2001) and eating pathology, including the reduction of binge episodes in 89% of the DBT group at the end of the intervention (Telch, Agras, and Linehan, 2001). At a 6-month follow-up, 56% of the DBT sample had managed to abstain from binging (Telch et al. 2001).

Problematic eating behaviors like binge or emotional eating increase the risk of developing obesity and vice versa; however, those conditions may appear separately (APA, 2013; Zeeck, Stelzer, Linster, Joos, and Hartmann, 2011). Nevertheless, its worthy to mention that, for individuals with obesity and problematic eating behaviors, the standard dietary treatment associated with physical exercise may not be effective, triggering the opposite effect, since food restriction increases the tendency to eat motivated by emotions (Macht, 2008). In addition, breaking diet patterns can generate stress, which can be regulated again through emotional eating, creating a vicious cycle (Macht and Simons, 2011). For these reasons, eating behaviors related to the regulation of negative emotional states has been indicated as a vital issue for the effective treatment of weight loss and maintenance (Levitan and Davis, 2010).

Some pilot studies have investigated the effect of DBT skills training on problematic eating behaviors and binge eating in individuals with obesity, suggesting that skills training may reduce emotional eating (Roosen, Safer, Adler, Cebolla, and Van Strien, 2012), improve mood, and decrease binge eating in individuals with obesity who regained weight after bariatric surgery (Himes et al. 2015). The treatment has also been found to decrease binge eating in patients seeking weight management services (Mushquash and McMahan, 2015). Additionally, a study by Soleimani and colleagues (2012) found positive effects on the part of a DBT skills group on self-esteem in women with binge eating disorder [BED] seeking treatment at a weight loss clinic, as compared to a waitlist control condition. Cancian et al. (2017) found that an adapted DBT skills training intervention may decrease binge and emotional eating in individuals with obesity post-treatment as compared with waitlist controls, although the large and medium effect sizes were not statistically significant. The current study utilizes data from this pilot trial and further analyzes the effects on the sample at follow-up.

Regarding the stability of the results found with DBT to problematic eating behaviors, a randomized controlled trial [RCT] conducted by Safer et al. (2010) compared group DBT sessions to active comparison group therapy [ACGT] and monitored the effects upon 3-, 6-, and 12-month follow-up. Individuals with BED (*n* = 101) were randomly assigned to the DBT-BED (*n* = 50) or ACGT (*n* = 50) groups. The results showed that the abstinence rate for the DBT-BED group was 64% post-treatment, 51% at the 3-month follow-up, 52% at the 6-month follow-up, and 64% by the 12-month follow-up. Compared to the ACGT group, in the DBT-BED group, abstinence was achieved more rapidly. However, significant differences did not persist at follow-up, which suggests that both treatments were effective and emphasize the need for more studies controlling the effects within time (Safer, Robinson, and Jo, 2010). Research also indicates that binge eaters with certain profiles respond better to DBT than to active control conditions, especially individuals with early-onset dieting or overweight and those with avoidant personality disorders (Robinson and Safer, 2012).

Given that the rationale behind DBT is to create and generalize alternative skills with which to deal with problematic behaviors (Linehan and Wilks, 2015), it is also necessary for researchers to investigate whether DBT skills training can develop adaptive skills and more effective behaviors, as well as whether changes in these skills are related to outcomes. Mindfulness skills are the core of the treatment program, and their main objective is to increase the awareness of the present moment, without critical self-consciousness or judgment, replacing mindless eating, binge and emotional eating, and other problematic eating behaviors. Mindfulness is also the foundation of other skills because one must be mindful to decide what is reasonable behavior and what is not (Safer et al. 2009).

Mindful eating can be defined as a non-judgmental awareness of the physical and emotional sensations associated with eating (Framson et al. 2009). Dialectical behavior therapy conceptualizes mindful eating as participating in the present moment by using the skills of observing, describing, and participating (Safer et al. 2009). According to Wisniewski, et al. (2007) the goal of DBT Skills Training for problematic eating patterns is to find the *path to mindful eating*. For that, it is necessary that individuals decrease (a) problematic eating behaviors, like binge and emotional eating, (b) mindless eating, (c) cravings, urges, and preoccupation with food, (d) capitulating, (e) apparent irrelevant behaviors, like not weighting or body checking. And most importantly, along with that, it is essential that the individual learn and increase skillful emotion regulation behaviors, applying mindfulness skills, emotion regulation skills, and distress tolerance skills. Following the path to mindful eating can naturally lead to healthy weight and increase overall wellness (Wisniewski, Safer, and Chen, 2007).

Mindfulness is also related to the concept of intuitive eating, which describes the tendency of an individual to be observant of his or her body signals and emotions and to eat according to physical hunger and satiety cues rather than emotions and rigid rules about the “right” and “wrong” types of food. Intuitive eating encourages the pleasure derived from eating, along with a healthy relationship with food, mind, and body, instead of engaging in reactive maladaptive eating behaviors. Therefore, intuitive eating encompasses the mindfulness components of acceptance and awareness (Tribole and Resch, 2012). A study by Duarte et al. (2016) found that intuitive eating significantly moderated the relationship between negative affect and binge eating, suggesting that increased levels of intuitive eating may buffer the effects of depressive symptoms on binge eating. Mindful eating and intuitive eating have been found to be inversely associated with binge and emotional eating (Katterman, Kleinman, Hood, Nackers, and Corsica, 2014). As those concepts of mindful and intuitive eating represent adaptive eating behaviors, related especially with present moment awareness, these variables are naturally inversely associated with eating triggered by emotions or lacking awareness, like in binge or emotional eating. However, the effect of an DBT skills training-based intervention and time still need to be examined in intuitive eating and mindful eating. Moreover, if DBT skills training leads to the path of mindful eating (Wisniewski, Safer, and Chen, 2007) the mediational role of mindful eating needs to be investigated.

Therefore, even though there is evidence that DBT is effective in addressing disordered eating behaviors and other related problems (Bankoff, Kappel, Forbes, and Pantalone, 2012), this study fills in an important gap, as there is still a need to investigate whether the effects of DBT skills training are lasting, the effect on adaptive eating behaviors in the long term, and whether changes in mindful eating contribute to improvements in eating behavior. For that purpose, this study implements a one group pre-experimental design to analyze participant’s problematic and adaptive eating behaviors before and after a DBT-based skills training intervention, conducting secondary analyses on the sample utilized by Cancian et al. (2017). Problematic and adaptive eating measures were used as main outcomes, and it was tested whether changes on these outcomes were associated with the intervention at 3- and 8-month follow-up and whether they were mediated by mindful eating.

## Material and methods

In the present study, the authors conducted secondary analyses of a pilot study of a DBT-based skills training intervention for individuals with problematic eating behaviors and obesity. The main aim of the current study is investigating changes in problematic and adaptive eating behaviors at post-test, 3-, and 8-month follow-up. Problematic eating behaviors are defined as binge eating and emotional eating, and adaptive eating behaviors are defined as mindful eating and intuitive eating, and all variables were measured at multiple points: extended baseline, pre-test, post-test, 3-, and 8-month follow-up. By including assessment of outcome at multiple points, this study also investigated (i) measure’s test-retest reliability, (ii) if mindful eating mediated changes in the variables across time. A summary of key study variables and procedures is included below; please see Cancian et al. (2017) for further details.

### Intervention protocol

The intervention was a DBT-based skills training group intervention that included skills from the mindfulness, emotion regulation, and distress tolerance modules. Table 1 displays the intervention according to the template for intervention description and replication (TIDieR) checklist and guide (Hoffmann et al. 2014), and details intervention rationale, adaptations, where, how and when it was delivered, and how structured and well it was carried out. The intervention was adapted from the second edition of the original DBT skills training protocol developed by Linehan (2015) and based on the DBT skills training protocol adapted to bulimia and binge eating by Safer et al. (2009). The skills were translated to Portuguese and analyzed by three judges with official DBT training, reaching 92% of concordance between the judges about adequacy and consistency with the original DBT concepts (Cancian et al. 2017). The present study protocol with the modules, skills taught, and mindfulness practices is described in Table 2. For further details about the adaptation process, please refer to Cancian et al. (2017).

**Table 1:** Intervention description and replication (TIDieR) checklist

Item number	Item
Brief name	
1	Dialectical behavior therapy-based skills training group intervention for Brazilian individuals with obesity.
Why	
2	The rationale of DBT is that problematic behaviors are attempts to regulate intense emotional states and that these behaviors are harmful in the long term. The skills training aims to teach new behavioral skills that can replace dysfunctional behaviors. The main goal of the present intervention was to teach emotion regulation skills and thus help individuals manage their problematic eating behaviors and increase adaptive eating behaviors.
What	
3	The trainers utilized standardized audiovisual material to conduct the intervention and a structured theme agenda, both of which were developed to increase professional adherence to the protocol, regardless of the group. In each session, participants received printed material to follow during the skills class and printed material that served as homework. The printed materials utilized in the intervention by the leaders to conduct the sessions and given to the participants are both available in the online appendix.
4	Group sessions began with mindfulness practice (10 min) and then moved to a homework review from the previous session (30 min), continued with a brief break (5 min), and ended with teaching new material (1 h and 15 min) according to the standard DBT group format.
Who provided	
5	Two psychologists lead the intervention, one who attended a 10-day intensive DBT workshop conducted by a certified senior DBT trainer (author 2), and one who received a 200-h DBT training conducted by two experienced DBT trainers (author 1). The researchers trained three groups each, and for each group, a different co-leader was chosen from the consultation team. The co-leaders were trained by the researchers, participated in a reading group 6 months prior to the intervention, and went through the intervention themselves (conducted by the researchers).
How	
6	Face-to-face adapted DBT skills training was conducted by two trainers in groups, and each group included a maximum of nine participants.
Where	
7	The participants were recruited at the university, and the sessions were carried out in the same location. The infrastructure included a classroom, chairs, a projector, a computer (for slides presentation), and air conditioning.
When and how much	
8	The intervention was composed of 2-h sessions conducted twice a week. The sessions were scheduled according to participants’ preferences (in the morning, in the afternoon, or at night). Once a participant chose a group, he or she could not change to another time or group. In total, six groups received the intervention. Two groups were trained in the morning, from 10 am to 12 am; two groups were trained in the afternoon, from 15 pm to 17 pm; and two groups were trained at night from 19 pm to 21 pm
Tailoring	
9	The intervention was adapted to Brazilian Portuguese and to the population with obesity.
Modifications	
10	The intervention was not modified during the study.
How well	
11	A consultation team was formed to increase adherence to the model. Intervention fidelity was discussed weekly in the consultation team meetings. The consultation team was formed by seven professionals who participated as leaders or co-leaders of the intervention. The weekly consultation meeting aimed to coach team members who fell outside of the DBT model used for the intervention reduce burnout by offering support and validation to the trainers, and provide feedback on the conducting of the intervention.
12	The intervention was generally delivered as planned. However, some leaders had difficulties regarding time management. In addition, participants tended to provide life examples unrelated to eating contexts, so trainers had to provide additional examples to explain the use of skills related to eating behaviors.

**Table 2:** Adapted DBT skills training intervention (as shown in Cancian et al. 2017)

Session	Module	Selected handouts and worksheets	Mindfulness exercise and skills taught
1	Orientation and mindfulness	General handout 1	Mindfulness: breath
		Mindfulness handout 1A, 3 e 4 mindfulness worksheet 4A	Goals of skills training
			Mindfulness definitions
			Wise mind—states of mind
			Taking hold of your mind—“what” skills
2	Mindfulness	Mindfulness handout 5	Mindfulness: find your lemon
		Mindfulness worksheet 2 e 5A	Taking hold of your mind—“how” skills
3	Emotion regulation	Emotion regulation handout 1, 3, 4, 5 e 6	Mindfulness: wise mind—stone flake on a lake
		Emotion regulation worksheet 2	Goals of emotion regulation
			What emotions do for you
			What makes it hard to regulate your emotions
			Model for describing emotions
			Ways to describe emotions
4	Emotion regulation	Emotion regulation handout 8, 8A	Mindfulness: mindful participation—improvisation
		Emotion regulation worksheet 5	Check the facts
			Examples of emotions that fit the facts
5	Emotion regulation	Emotion regulation handout 9, 10, 12 e 13	Mindfulness: noticing urges
		Emotion regulation worksheet 7 e 8	Opposite action and problem solving—deciding which to use
			Opposite action
			Problem solving
			Reviewing opposite action and problem solving
6	Emotion regulation	Emotion regulation handout 15, 16, 17 e 18	Mindfulness: noticing sensations
		Emotion regulation worksheet 9	Accumulating positive emotions—short term and long term
			Pleasant events list
			Values and priorities list
7	Emotion regulation	Emotion regulation handout 19 e 20	Mindfulness: raisin
		Distress tolerance handout 8	Build mastery and cope ahead
		Emotion regulation worksheet 9 e 16	Taking care of your mind by taking care of your body
			Self-soothing
8	Distress tolerance	Distress tolerance handout 7 e 9	Mindfulness: noticing sounds
			Distracting
			Improving the moment
9	Distress tolerance	Distress tolerance handout 5, 11 e 11B	Mindfulness: body scan
		Distress tolerance worksheet 3 e 9	Pros and cons
			Radical acceptance
			Practicing radical acceptance step by step
10	Review		Mindfulness: wise mind—focus on your breath

### Participants

**Fig. 1:**
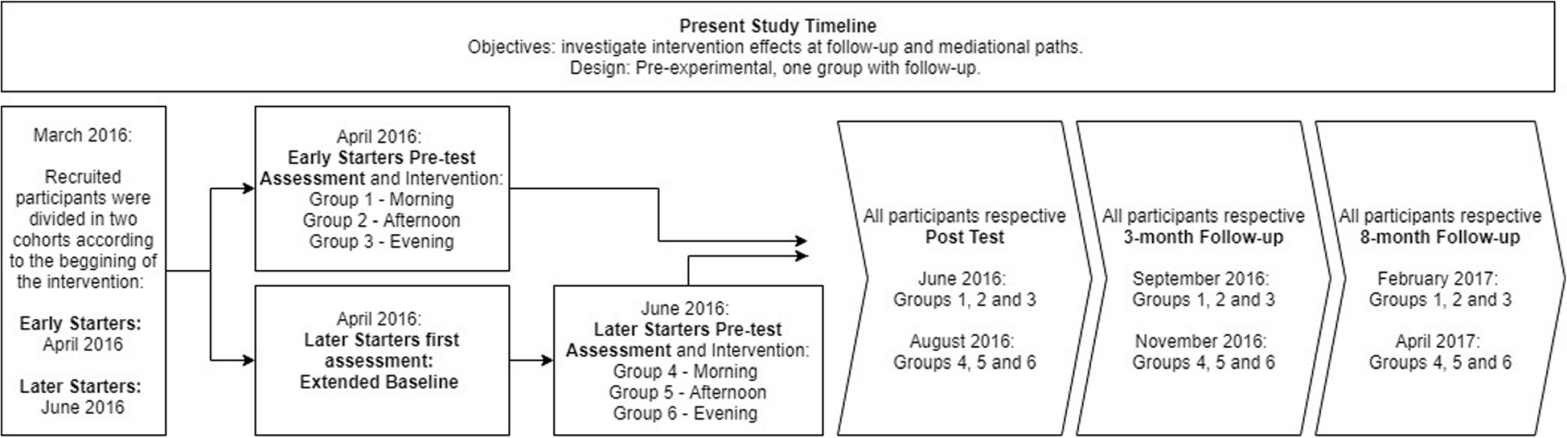
Present study timeline and procedures. Each rectangle represents procedures in the study’s timeline

The inclusion criteria of the present study consisted of completing a 10-session intervention protocol. The sample is derived from the study of Cancian et al. (2017) constituted by adult individuals (18 to 59 years), with a Body Max Index (BMI) greater than 30 and at least 8 years of formal schooling. For the present study, the subjects completed assessments in multiple points like described on Fig. 1.

The sample was divided into two cohorts, early starters and later starters; each cohort was offered morning, afternoon, or evening groups. Measurements were delayed accordingly in order to maintain an equivalent measurement schedule. The first pre-test measurement performed for participants that were later starters was used as an extended baseline, in order to compare measurements stability within time. The second measurement taken from those participants immediately before starting the intervention was used as a regular pre-test measure, like displayed on Fig. 1. Follow-up assessments were performed on a digital survey platform (Qualtrics). Data from participants were pooled for all analyses in the current study, configuring one group.

Authors are adhering to the full disclosure procedures that allow accurate replications (Peters, Abraham, and Crutzen, 2015). The present study protocol and datasets are public in the platform Open Science Network.

### Instruments

Except for sociodemographic information (collected at baseline only), all questionnaires were administered at baseline, post-treatment, and at 3 and 8 months following treatment, as depicted in Fig. 1.

#### Measures of problematic eating behaviors

##### Emotional Eating Scale (EES; Arnow, Kenardi, and Agras, 1995)

The scale has 25 items with three subscales that target feelings in the domains of *Anger*, *Anxiety,* and *Depression*. Respondents are asked to rate the urge to eat when experiencing different types of feelings, using a 5-point Likert scale ranging from “1 - no desire to eat” to “5 - an overwhelming urge to eat.” Higher total scores indicate greater levels of overall emotional eating. The scale’s internal consistency ranged from α = .92 to α = .98 on the present sample, and a coefficient omega of 0.92, with good test-retest stability (ICC = .84)

##### Binge Eating Scale (BES; Gormally, Black, Daston, and Rardin, 1982; Freitas, Lopes, Coutinho, and Appolinario, 2001 for the Brazilian version)

This scale is a 16-item self-report measure with three or four options of increasing severity regarding eating behaviors and cognitions. It can be used to assess binge eating symptomatology and behaviors. Higher scores indicate more severe binge eating attitudes. The BES also allows the discrete classification of binge eating severity based on participant scores: *mild/no binge eating* (0–17), *moderate binge eating* (18–26), and *severe binge eating* (27–46). The differences between moderate and severe binge eating are the degree and frequency of the loss of control over eating urges and the severity of the emotional consequences of overeating. The BES showed high internal consistency scores in the present study, with alphas ranging from α = .85 to α = .92, and ordinal omega of 0.85, as well as a test-retest stability of ICC = .60.

#### Measures of adaptive eating behavior

##### Intuitive Eating Scale-2 (IES-2; Tylka and Kroon Van Diest, 2013)

The scale is a 23-item self-report that includes four dimensions. The *Eating for Physical Rather than Emotional Reasons* dimension represents individuals’ patterns of eating, including whether eating is driven by physical hunger or to cope with emotional distress, such as anxiety, loneliness, or boredom. The *Unconditional Permission to Eat* dimension reflects individuals’ willingness to eat when hungry and tendency to refuse labelling foods as forbidden, good, or bad based on arbitrary criteria or strict dietetic norms. The *Reliance on Hunger and Satiety Cues* dimension represents individuals’ reliance on their internal hunger and satiety cues to guide eating behavior. Finally, the *Body-Food Choice Congruence* dimension reflects the tendency of individuals to make food choices that are aligned to their health and body functioning, choosing foods that enable body performance and taste good. Items are rated on a 5-point Likert scale ranging from 1 (strongly disagree) to 5 (strongly agree). Higher scores indicate greater levels of intuitive eating. The IES-2’s internal consistency for the sample ranged from α = 0.61 to α = 0.74, with ordinal omega of 0.58. The stability level was low (ICC = .29).

##### Mindful Eating Questionnaire (MEQ; Framson et al. 2009)

The self-report questionnaire is composed of 28 items that assess 5 domains of mindful eating. The *disinhibition* domain assesses the inability to stop eating even when full. The *external cues* domain reflects eating in response to external environmental cues. The *awareness* domain assesses the awareness of and attention paid to the moment of eating and the perception of the effects of food on the senses. The *emotional response* domain reflects eating that is triggered by negative emotional states. Finally, the *distraction* domain assesses how much the individual focuses on other activities while eating. The questionnaire uses a Likert-type 4-point scale that ranges from “never / rarely” to “almost always / always”. The evidence regarding internal consistency in the sample was not conclusive, with alphas ranging from α = .74 to α = .88, and very high test-retest stability (ICC = .89), while coefficient omega was very low (0.25).

### Data analysis

The instruments reliability was assessed by determining alpha and hierarchical omega coefficients. These were obtained by using the *userfriendlyscience* R package (Peters, 2018). Test-retest stability was assessed via the intraclass correlation coefficient implemented in the *psych* package (Revelle, 2018), computed with the first two measures—baseline and pre-test.

Following that, an initial data exploration was held, aiming at providing detailed insights into the observed courses of the outcome variables. The means and standard errors were plotted for each variable over time, using the within-subject standard error formula implemented in the *Rmisc* package (Hope, 2013), which is based on the work of Morey (2008). Plots were generated using the *ggplot2* package (Wickham, 2009).

Following the data exploration, the effects of time and the intervention on each of the relevant outcomes were tested. Adaptive and problematic eating outcomes were analyzed using a linear mixed-models approach. This approach is advantageous because of its ability to account for within-subject correlation and include subjects with missing data. Using this approach, all subjects could be included in the analysis, regardless of having some missing data points. Thus, our approach represents an intention-to-treat approach (Chakraborty and Gu, 2009).

In order to test our various hypotheses, three alternative models were assessed for model fit based on the Akaike information criterion (AIC). All models included the subject identification variable as a random factor. The first model tested the effect of time, while the second tested the effect of the intervention on the outcomes, and the third tested both the effect of the intervention and the interaction between time and the intervention. The first model tests the hypothesis that any change in outcomes is due to natural growth, while the second tests whether the intervention is an explanatory factor for the observed changes, and the third tests whether the effects remain at follow-up or decrease with time.

The analysis was conducted using the *lme4* (Bates, Mächler, Bolker, and Walker, 2015) package for the R statistical programming framework (R Core Team, 2017). As an additional measure of model fit, conditional and marginal coefficients of determination were estimated using the MuMIN package (Bartoń, 2017). These are based on the method proposed by Nakagawa and Schielzeth (2013), in which the marginal r-squared value (*R*^2^_m_) represents the variance explained by the fixed factors in the model and the conditional r-squared value (*R*^2^_c_) represents the total variance explained by both the fixed and random effects in the model. The confidence intervals and *p* values for the unstandardized regression coefficients were computed using the Wald approach, as implemented by the sjstats package (Lüdecke, 2017).

After determining whether there was a detectable effect on the part of the intervention or time, a mediation analysis was conducted using a path analysis. This analysis was performed in the medmod R package, as implemented by Jamovi software (Jamovi Project, 2018). The path model was based on the hypothesis that an increase in mindful eating would explain most of the effects of the intervention on binge eating, emotional eating, and intuitive eating. According to this hypothesis, a statistically significant positive effect on the part of the intervention on mindful eating is expected (*a* pathway). Effects on the part of mindful eating on adaptive and problematic eating outcomes (*b* pathway) are also expected to be statistically significantly negative for binge and emotional eating and positive for intuitive eating. Given that mindfulness is not the only active component of the DBT skills training intervention, only partial mediation is expected.

Thus, the occurrence of significant residual direct effects on the part of the intervention (*c* pathway) was plausible in this model. The confidence intervals for the direct, indirect, and total effects on each outcome, as well as for path estimates, were computed using the bootstrap method with 1000 re-samplings.

## Results

### Sample characteristics

The sample consisted of 121 adults, 95% of which were women (*n* = 115), with a mean age of 38.49 years (SD = 10.54) and an average BMI of 38.56 kg/m2 (SD = 5.98). Regarding marital status, most participants were married or common-law married (57.9%, *n* = 70), followed by those who were single (27.3%, *n* = 33) and divorced (9%, *n* = 12). One participant was widowed, and 4.1% declared some other status (*n* = 5). Most of the sample had at least some post-secondary education (47.1%, *n* = 57) or had completed secondary education (26.4%, *n* = 32). Around 15% had less than a secondary education (*n* = 18), and 11.6% had completed graduate education or higher (*n* = 14). Roughly 69% of participants declared an income of one to two Brazilian minimum wages (*n* = 84), 23.1% declared an income of three to four minimum wages (*n* = 28), and a minority declared an income of five or more minimum wages (5.8%, *n* = 7). More than a half of the sample was employed (*n* = 72), and fewer were homemakers (12.4%, *n* = 15) or students (10.7%, *n* = 13). Four participants reported being retired (3.3%), and 14% reported having other occupations (*n* = 17).

At baseline, around a half of participants reported a health problem (51.2%, *n* = 62), and a similar percentage had already received psychological or psychiatric treatment (54.5%, *n* = 66). The majority had received weight loss treatments in the past (72.7%, *n* = 88), and a similar percentage was not currently exercising (62%, *n* = 75).

### Eating behavior outcomes

**Fig. 2:**
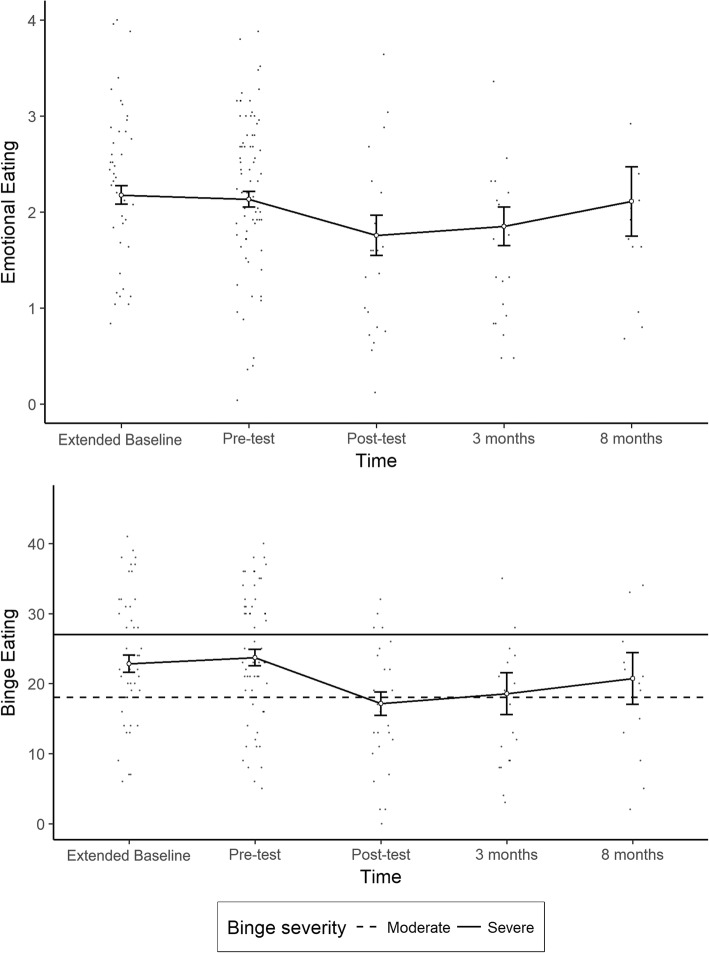
Trajectories of problematic eating behaviors across time. Dots represent individual scores for each timepoint. White dots and error bars indicate means and 95% confidence intervals for each timepoint. On the binge eating graph, the horizontal lines represent cutoff scores for moderate binge eating (dashed line) and severe binge eating (solid line)

**Fig. 3:**
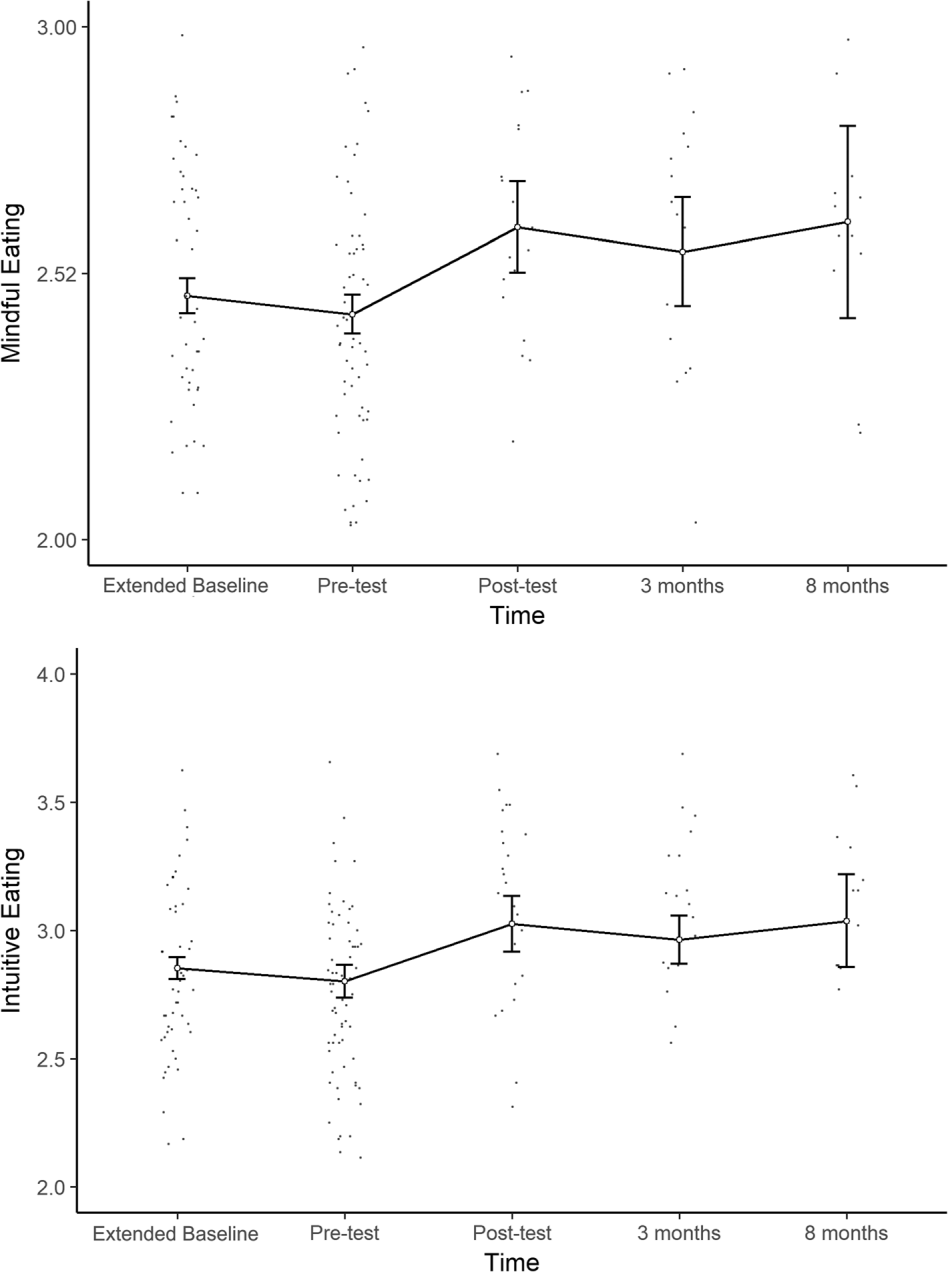
Trajectories of problematic eating behaviors across time. Dots represent individual scores for each timepoint. White dots and error bars indicate means and 95% confidence intervals for each timepoint

The trajectories of the outcomes over time show an improvement in adaptive eating and a decrease in problematic eating behaviors directly after the intervention. Moreover, the data seem to be compatible with the hypothesis that the effects of the intervention remain stable at follow-up. Nonetheless, the plots do show a slight tendency towards returning to baseline values at the 8-month follow-up. Figures 2 and 3 show the trajectories of each outcome variable across all five timepoints, with theses results bein shown numerically in Table 3, while Table 4 shows the results of the linear mixed-model analysis. In the following, the results are presented in two groups: *problematic eating behaviors* and *adaptive eating behaviors*.

**Table 3:** Outcomes regarding adaptive and problematic eating behaviors after intervention, 3- and 8-month follow-up

Variable	Extended baseline	*n*	Pre-test	*N*	Post-test	*n*	3-month follow-up	*n*	8-month follow-up	*n*
Emotional eating—EES total	2.24 (2–2.48)	50	2.16 (1.96–2.36)	71	1.47 (1.1–1.84)	25	1.56 (1.21–1.91)	19	1.73 (1.36–2.1)	12
Intuitive eating—IES-2 total	2.77 (2.67–2.87)	51	2.74 (2.66–2.82)	67	3.13 (2.97–2.79)	26	3.15 (2.97–3.33)	19	3.21 (3.01–3.41)	14
Mindful eating—MEQ total	2.43 (2.35–2.51)	51	2.41 (2.33–2.49)	67	2.63 (2.47–2.79)	26	2.68 (2.54–2.82)	19	2.7 (2.54–2.86)	14
Binge eating—BES total	24 (21.41–26.59)	48	24.33 (21.94–26.52)	61	16.44 (12.85–20.03)	25	16.06 (11.96–20.16)	18	18.62 (13.33–23.91)	13

Note: Mean (95% Confidence Interval); *EES* Emotional Eating Scale, *IES-2* Intuitive Eating Scale, *MEQ* Mindful Eating Questionnaire, *BES* Binge Eating Scale

**Table 4:** Linear mixed-model analysis results for adaptive and problematic eating behaviors

	EES—emotional eating			BES—binge eating			MEQ—mindful eating				IES-2—intuitive eating	
	*B*	CI	*P*	*B*	CI	*P*	*B*	CI	*p*	*B*	CI	*p*
Fixed parts												
(Intercept)	2.19	2.03–2.35	< .001	24.39	22.57–26.21	< .001	2.42	2.36–2.49	< .001	2.74	2.67–2.81	< .001
Int	− 0.54	− 0.75 to − 0.33	< .001	− 8.28	− 10.58 to −5.98	< .001	0.25	0.16–0.33	< .001	0.37	0.26–0.47	< .001
Random parts												
σ^2^	0.289			32.824			0.050				0.085	
τ_00, ID_	0.417			51.855			0.060				0.054	
N_ID_	105			95			100				100	
ICC_ID_	0.590			0.612			0.546				0.387	
Observations	177			165			177				177	
*R*^2^_m_/*R*^2^_c_	.083/.624			.154/.672			.111/.596				.176/.495	

Note: *B* unstandardized regression coefficients, *CI* 95% confidence intervals are computed using the Wald method, *p p* values obtained using Wald statistic, *ICC* intraclass correlation coefficient, *EES* Emotional Eating Scale, *IES-2* Intuitive Eating Scale, *MEQ* Mindful Eating Questionnaire, *BES* Binge Eating Scale

### Problematic eating behaviors

All of the problematic eating behaviors assessed showed improvement after the intervention, including significant reductions in emotional eating and binge eating. Figure 2 shows the reference lines for the binging severity ratings based on the BES’s recommended interpretation. This figure shows that directly after the intervention, the average scores were below the threshold for moderate binging and remained at this level after 3 months, with an upwards trend appearing at follow-up. Nonetheless, this trend could not be corroborated by the linear models, because the model with the interaction term was not superior to the simpler models for any of the variables.

The intervention effect followed the expected direction for all problematic eating outcomes, as can be seen by the negative regression coefficients for binge eating (*B* = − 8.28, *p* < 0.001) and emotional eating (*b* = − 0.54, *p* < 0.001). The amount of variance in the binge eating scores that was explained by the intervention was around 15%, with 67% of the variance being explained by the total model, which includes within-subject variance (*R*^2^_m_ = 0.154, *R*^2^_c_ = 0.672). The amount of variance explained in emotional eating scores was 8.3%, with the entire model explaining 62% of the total variance in this outcome (*R*^2^_m_ = 0.083, *R*^2^_c_ = 0.624).

### Adaptive eating behaviors

The adaptive eating outcomes showed rather similar trajectories, only in the inverse direction. The plots show noticeable improvements after the intervention, both in intuitive eating and mindful eating, with no change between the pretest measures. No trend towards the baseline could be detected for either of the adaptive eating measures.

The linear mixed-model analysis shows a statistically significant effect on the part of the intervention on both intuitive eating (*b* = 0.37, *p* < 0.001) and mindful eating (*b* = 0.25, *p* < 0.001). The intervention explained approximately 18% of the total variance in intuitive eating and 11% of that in mindful eating, while the entire model explained 49% of the total variance in intuitive eating scores (*R*^2^_m_ = 0.176, *R*^2^_c_ = 0.495) and 59% of the variance in mindful eating scores (*R*^2^_m_ = 0.111, *R*^2^_c_ = 0.596).

### Mediation analysis

**Fig. 4:**
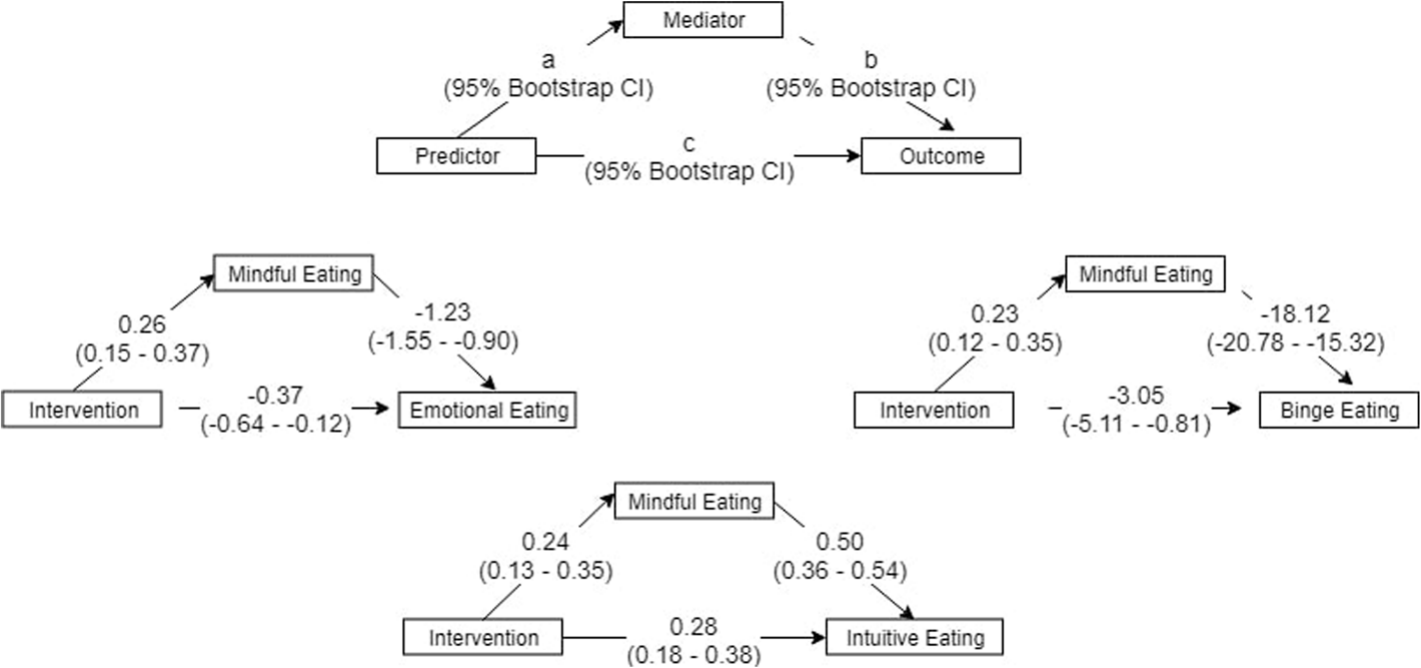
Path models for direct effects and mediation estimates. Each model shows relationships among three variables: a predictor, a mediator, and an outcome. The intervention is the predictor on all three models, and mindful eating is the mediator. The outcomes are emotional eating, intuitive eating, and binge eating. Numbers on the arrows indicate the path coefficients for the effect of the predictor on the mediator (*a* path), effect of the mediator on the outcome (*b* path), and the effect of the predictor on the outcome (*c* path). The 95% confidence intervals for the path coefficients were calculated with the bootstrap method and are shown in parenthesis below each coefficient

The results from the path analysis show that the data were compatible with the hypothesis that mindful eating scores partially mediate the effects of the intervention on adaptive and problematic eating scores. The path coefficients and 95% bootstrap confidence intervals are displayed in Fig. 4. In addition to the effects of the intervention on the mediator, and the effects of the mediator on outcomes, direct effects of the intervention on the outcome variables were also present in all models. Thus, it can be inferred that even though mindful eating explains a significant portion of the effect of the intervention, other variables may be needed to fully understand the mechanisms via which the DBT skills training produces its effects.

The estimates and confidence intervals for the direct, indirect, and total effects of the intervention are displayed in Table 5. Both the direct and indirect effects were statistically significant for all outcome variables. While indirect effects were larger than direct effects for binge eating scores, the opposite was true for the emotional eating and intuitive eating outcomes. The direction of the observed effect was coherent with that expected based on the theory that underlies the model construction. The direct effects of the intervention on the mediator were positive, while the direct effects of the intervention on the outcomes were negative for problematic eating and positive for adaptive eating. The indirect effect was also positive for adaptive eating and negative for problematic eating.

**Table 5:** Mediation estimates with 95% bootstrap confidence interval

95% confidence interval								
Effect	Label	Estimate	SE	Lower	Upper	Z	*p*	% mediation
Binge eating								
Indirect	*a* × *b*	− 4.22	1.08	− 6.43	− 2.13	− 3.89	< 0.001	58.03
Direct	*C*	− 3.05	1.11	− 5.11	− 0.81	− 2.75	0.006	41.97
Total	*c* + *a* × *b*	− 7.27	1.46	− 10.19	− 4.38	− 4.99	< 0.001	100.00
Emotional eating								
Indirect	*a* × *b*	− 0.32	0.08	− 0.47	− 0.18	− 4.17	< 0.001	46.28
Direct	*C*	− 0.37	0.13	− 0.64	− 0.12	− 2.77	0.006	53.72
Total	*c* + *a* × *b*	− 0.69	0.14	− 0.96	− 0.41	− 4.99	< 0.001	100.00
Intuitive eating								
Indirect	*a* × *b*	0.12	0.03	0.06	0.19	3.79	< 0.001	30.09
Direct	*C*	0.28	0.05	0.18	0.38	5.32	< 0.001	69.91
Total	*c* + *a* × *b*	0.40	0.06	0.28	0.52	6.74	< 0.001	100.00

Note: *a* path estimate for the effect of the intervention on mindful eating, *b* path estimate for the effect of mindful eating on outcome, *c* path estimate for the effect of the intervention on outcome

## Discussion

The results of the present study indicate improvements in patterns of eating, along with decreased problematic eating behaviors, after a DBT-based skills training intervention. These improvements were sustained at follow-up. One of the dimensions of adaptive eating that seems to have improved was intuitive eating. Intuitive eating encompasses one’s abilities to be aware of physical cues of hunger and satiety, using physical cues rather than emotional ones to decide when to eat, and making food choices that are congruent with bodily health needs rather than with arbitrary rules or diets (Tylka and Kroon Van Diest, 2013). Regarding this outcome, the model of the intervention explained 18% of the variance. The observed improvements in intuitive eating suggest that the contexts that influence eating behavior may have changed, becoming more related to internal cues and bodily functions and less dependent on mood, affect, and external rules. Other intervention studies that targeted mindfulness to tackle problematic eating show results that are consistent with these (Dalen et al. 2010; Kristeller and Hallett, 1999).

In line with these results, mindful eating was also found to be improved after the intervention. For mindful eating, the intervention model explained 11% of the variance. The mindful eating domain assesses an individual’s adaptive abilities to stop eating when full, refrain from eating in response to emotional states, be aware of environmental cues that evoke eating behavior, notice mindless eating, and be aware of the effects of food on the senses (Framson et al. 2009). Therefore, the pattern defined by better mindful eating scores, combined with lower measures of emotional eating, that occurred after the intervention is theoretically consistent with the rationale underlying the proposal of DBT as an intervention to tackle problematic eating. It is expected that individuals could learn other skills with which to control their emotions rather than use eating to cope. Also, the results support the hypothesis that mindfulness skills help to develop a better awareness of the context surrounding eating behaviors, including the act and taste of eating (Safer et al. 2009).

Safer et al. (2009) suggest that turning to food in response to emotional dysregulation often is automatic and that mindfulness skills can empower the individual to break the link between emotions and automatic eating. When practicing mindfulness, the idea is to acknowledge and fully accept any emotional state, as it arises and passes away, without trying to avoid or escape by mindlessly eating or binging (Safer et al. 2009). This is consistent with the improvements found in this study given that after the intervention, individuals were less prone to turn to food when emotional than they were before the intervention and also were more aware of the various aspects of eating.

The results of the mediation analysis were compatible with the hypothesis that mindful eating scores mediate the effects of the intervention on measures of problematic and adaptive eating. Because DBT is an intervention that in addition to developing mindfulness skills, also promotes the acquisition of more effective emotion-regulation strategies, it was hypothesized that the reduction in emotional eating frequency and binge eating severity would not be fully explained by an increase in mindfulness skills or mindful eating alone. In the rationale for DBT, it is assumed that a broadening on the individual’s emotional regulation repertoire will also affect the outcome. Indeed, the data show that decreases in emotional eating and binge severity were only partially mediated by increases in mindful eating following the intervention. Other studies using DBT interventions for various kinds of emotional problems show that measures of DBT skills use mediate outcomes (Neacsiu, Eberle, Kraemer, Wiesman, and Linehan, 2014). Also, it has been suggested that emotion regulation is a mechanism of change in acceptance-and-mindfulness-based therapies, a class of therapies to which DBT belongs (Gratz and Tull, 2010). Thus, it would be useful to include measures of DBT skills use and emotion regulation as additional mediators that could help in explaining the effects of DBT on eating-related problems.

This study showed that changes in mindful eating scores after a DBT-based skills training are associated with improvements in measures of problematic and adaptive eating behavior. Mindfulness, as it is addressed in DBT programs, includes a complex set of components that go beyond what is conceptualized as mindful eating. Mindfulness skills are at the core of the treatment program, and their main goal is to increase awareness of the present moment without self-consciousness or judgment, replacing mindless eating, binging, emotional eating, and other problematic eating behaviors. Mindfulness is also the foundation of other skills because one must be mindful to decide what is reasonable behavior and what is not. Also, being aware of one’s body and eating sensations may help to break the cycle of binge and emotional eating (Safer et al. 2009). Mindful eating, however, is only one aspect of mindfulness that relates to the context of eating. Other mindfulness traits have been shown to affect eating behavior independently of mindful-eating-specific training (Jordan, Wang, Donatoni, and Meier, 2014). Therefore, future studies that also measure other facets of mindfulness may be useful in elucidating the intervention’s mechanisms of change by isolating the components of mindfulness that are most relevant to bringing about changes in eating behavior.

The influence of mindful eating on actual eating behavior may be mediated by emotional reactivity (Beshara, Hutchinson, and Wilson, 2013). This is in line with what is proposed in DBT skills training, in which mindfulness skills are thought to be the base on which emotion-regulation skills and other relevant behaviors are grounded (Linehan, 2015). By simultaneously targeting emotion-regulation strategies and mindfulness abilities, DBT provides an effective framework for rapid behavioral change. Based on the findings of Beshara and collaborators (2013) and how they relate to those of the present study, it seems reasonable that future studies could focus on evaluating more complex mediational designs that include mindfulness and emotional reactivity within an expanded causal pathway that leads from the intervention to the outcome, including ecological moment assessments.

The considerable attrition observed throughout the study is an important limitation. Given a high rate of missing data, the possibility that the observed improvements are related to selection bias cannot be ruled out. It is possible that only those participants who benefited the most from the intervention remained in the study and agreed to participate until the final stage. The use of an intention-to-treat approach implemented via mixed-model analysis may, however, attenuate this bias. One way to test for selection bias is to determine whether baseline characteristics and participants’ outcomes predict dropout. Future research could also benefit from integrating the early assessment of known predictors of dropout, such as weight loss expectancy, social support, and practical issues, such as distance and financial difficulties (Moroshko, Brennan, and O’Brien, 2011).

The present study relied on self-report questionnaires for all outcomes. Even though these instruments are generally able to capture the occurrence and intensity of the behaviors of interest, they still rely on the participant’s ability to fully understand what is being asked, as well as his or her ability to accurately describe his or her own behavior in retrospect, which may be influenced by a myriad of factors, such as emotional state, literacy, and social desirability concerns. Also, the missing data regarding the initial stages due to incomplete questionnaires may be an indication of specific task-related difficulties, such as difficulties regarding the comprehension of the items or sustained attention throughout the assessment session.

Finally, one limitation of this study is that it does not compare the results with other interventions. As the available data do not provide full support for DBT’s superiority to CBT in the treatment of eating disorders (Linardon, Fairburn, Fitzsimmons-Craft, Wilfley, and Brennan, 2017), researchers should investigate the effects of DBT in the long term and compare it with other treatments, as well as identifying the specific profiles that may respond better to the intervention.

## Conclusions

In conclusion, the present study provides some evidence that a brief intervention focused on emotion regulation can be effective in increasing adaptive eating, especially mindful eating, and decrease problematic eating behaviors. The results are comparable to what has been found in longer interventions and were stable for the 3 months following the skills training. The evidence regarding stability at the 8-month follow-up, however, may require further investigation because the present study had high attrition rates at the 8-month follow-up assessment. The results provide preliminary support for the hypothesis that mindful eating may mediate the effect of DBT skills training on problematic and adaptive eating outcomes. Because this is the first investigation of the kind in a Brazilian sample of individuals with obesity, replications may be warranted to support broader conclusions. Future studies may advance the knowledge available on interventions targeted at emotional eating, binge eating, and mindful and intuitive eating by addressing the limitations of this study. Mainly, subsequent investigations should increase the accuracy with which outcomes are assessed. This may be accomplished by triangulating self-report measures with various assessment methods, such as ecological momentary assessment and experimental tasks. Additionally, more complex mediation models could be investigated, including other putative mediators and moderators of the effect of the intervention.

## Abbreviations

ACGT: Active comparison group therapy
BED: Binge eating disorder
BES: Binge eating scale
BMI: Body mass index
CBT: Cognitive behavioral therapy
cGSH: Guided self-help cognitive behavior therapy
DBT: Dialectical behavior therapy
EES: Emotional Eating Scale
IES-2: Intuitive Eating Scale-2
MEQ: Mindful Eating Questionnaire
*R*^2^_c_: Conditional *r*^2^
*R*^2^_m_: Marginal *r*^2^
RCT: Randomized clinical trial
